# Tonghua Liyan granules in the treatment of Laryngopharyngeal reflux disease with stagnation of phlegm and qi syndrome: a randomized, double-blind, placebo-controlled study

**DOI:** 10.3389/fphar.2024.1275740

**Published:** 2024-02-13

**Authors:** Yading Li, Mingxian Zheng, Yi Wang, Gaofan Xu, Yunyun He, Yue Wu, Xiao Wang, Yuyang Liu, Yujie Jiang, Xiaowen Liu, Yangyang Meng, Yixuan Yap, Shengliang Zhu, Cong He, Bingduo Zhou

**Affiliations:** ^1^ Yueyang Hospital of Integrated Traditional Chinese and Western Medicine, Shanghai University of Traditional Chinese Medicine, Shanghai, China; ^2^ Jinjiang Traditional Chinese Medicine Hospital, Fujian University of Traditional Chinese Medicine, Quanzhou, China

**Keywords:** laryngopharyngeal reflux disease, THLY granules, rabeprazole, integrated Chinese and Western medicine, efficacy evaluation

## Abstract

**Background:** Laryngopharyngeal reflux disease (LPRD) is an extraesophageal syndromic manifestation of gastroesophageal reflux disease (GERD). Despite the increasing incidence of and concern about LPRD, treatment with proton pump inhibitors (PPIs) is unsatisfactory. Here, LPRD was treated with Tonghua Liyan (THLY) granules in combination with PPIs to evaluate treatment efficacy and possible adverse reactions.

**Methods:** Seventy-six LPRD patients with stagnation of phlegm and qi syndrome (SPQS) were randomly divided into an experimental group and a control group. The experimental group received THLY granules combined with rabeprazole capsules. The control group received THLY granule placebo combined with rabeprazole capsules. A parallel, randomized, double-blind, placebo-controlled clinical trial was conducted with these two groups. The treatment cycle was 8 weeks. The reflux symptom index (RSI), clinical symptom score, salivary pepsin content, reflux finding score (RFS) and gastroesophageal reflux disease questionnaire (GerdQ) were used to evaluate clinical efficacy. The final efficacy rate was evaluated according to the RSI and clinical symptom score.

**Results:** Compared with those at baseline, all the indicators in the experimental group and control group significantly improved (*p* < 0.01). In terms of the RSI, clinical symptom score, and RFS, the experimental group had a higher degree of improvement (*p* < 0.05), and the overall efficacy rate was higher (*p* < 0.05). In terms of the salivary pepsin concentration and GerdQ, there was no significant difference between the test group and the control group (*p* > 0.05). Both groups of safety indicators showed no abnormalities and did not cause any allergic reactions in the body.

**Conclusion:** Compared with PPIs alone, THLY granules combined with PPIs are more effective in the treatment of LPRD patients with SPQS in terms of symptoms and signs. This combination treatment, because of its higher clinical efficacy and lack of obvious adverse reactions, is worthy of clinical promotion and further in-depth study.

**Clinical Trial Registration:** www.chictr.org.cn, identifier ChiCTR2100046614

## 1 Introduction

Laryngopharyngeal reflux disease (LPRD) is the reflux of gastric contents into the laryngeal cavity or even into the epiglottis, causing a sensation of foreign body in the throat, throat clearing, and hoarseness (Lechien et al., 2018). It is an extraesophageal syndrome manifestation of gastroesophageal reflux disease (GERD) (Katzka and Kahrilas, 2020). GERD symptoms are categorized as typical GERD, reflux chest pain, reflux cough, LPRD, reflux asthma, etc. LPRD can affect quality of life and lead to inflammatory reactions, mucosal damage, precancerous lesions, and even the formation of malignant tumors (Sasaki et al., 2019). Early diagnosis and intervention are therefore particularly important.

At present, there are no internationally harmonized diagnostic criteria for LPRD, and the lack of specific symptoms and laryngoscopic and gastroscopic signs makes it difficult to objectively diagnose and assess the extent of the disease clinically, resulting in misdiagnosis, mismanagement, overdiagnosis and treatment, and poor clinical efficacy. Therefore, many clinical practices and randomized controlled trials are needed for LPRD.

Proton pump inhibitors (PPIs) are the preferred treatment for LPRD in modern medicine, but treatment with PPIs fails to provide symptomatic relief in up to 40% of LPRD patients (Lechien et al., 2019). There is a high rate of relapse after discontinuation of the drug, while prolonged use of the drug produces more pronounced adverse effects. A survey showed that 21.1% of otolaryngologists estimated the prevalence of nonacidic LPRD and mixed LPRD to be 25.4% and 35.5%, respectively, of all LPRD patients (Lechien et al., 2020a). There is a lack of substantial clinical evidence for the effectiveness of PPIs in nonacidic LPRDs (Carroll et al., 2017; Lechien et al., 2020b). The efficacy of these treatments is less than optimal, so additional research has focused on drug development for nonacidic LPRD.

In the past 20 years, under the leadership of Professor Zhu Shengliang, the academic leader of our team, we have carried out clinical and experimental studies on typical GERD and extraesophageal syndromes at an early stage in China. In combination with the “Tong, Hua, Xuan, Ping” Differentiation and Treatment System of Ding’s Internal Medicine Chen Cunren Academic Ideology Research Base of Shanghai style Traditional Chinese Medicine (TCM), we creatively devised the “Tonghua Liyan (THLY) Decoction” (patent obtained: 202210102177.7), which is used in the clinic and has outstanding therapeutic effects. THLY, composed of 11 botanical drugs, 2 animal drugs and 1 mineral drug, is a combination of four well-known Chinese medicine formulas: Xuanfu Daizhe Decoction, Banxia Houpu Decoction, Gancao Jiegeng Shegan Decoction and Zuojin Wan (Table 1). Xuanfu Daizhe Decoction and Zuo Jin Wan, which are widely used for the treatment of GERD (Li et al., 2020; Li et al., 2022), have mainly anti-inflammatory effects and inhibit inflammatory responses through potential targets such as EGFR, IL-6, IL-1β, TNF-α and MCP-1 (Yu et al., 2018). Banxia Houpu Decoction has a good therapeutic effect on abnormal sensation in the throat and has been used to treat imagined plum pits in the throat in TCM (Bo et al., 2010). Gancao Jiegeng Shegan Decoction has various biological activities, such as analgesia, expectoration and cough suppression, and is mainly used for pharyngitis treatment (Li et al., 2022; Ding et al., 2022).

**TABLE 1:** Composition and dosage of THLY.

No.	Accepted name	Chinese name	Dosage per pack of granules (g)	Daily dose of decoction (g)
1^a^	*Inula japonica* Thunb. [Asteraceae; Inulae flos]	Xuanfuhua	1.30	12.00
2^a^	*Haematitum* [main component: Fe (2)O (3), (calcined)]	Duanzheshi	0.43	15.00
3^abc^	*Pinellia ternata* (Thunb.) Makino [Araceae; Pinelliae rhizoma]	Banxia	2.80	12.00
4^b^	*Magnolia officinalis* Rehder and E.H. Wilson [Magnoliaceae; Magnoliae officinalis cortex]	Houpu	0.80	12.00
5^b^	*Wolfiporia cocos* (F.A. Wolf) Ryvarden & Gilb. [Polyporaceae; Poria]	Fuling	0.60	15.00
6^b^	*Perilla frutescens* (L.) Britton [Lamiaceae] Perillae folium	Zisuye	1.50	12.00
7^ab^	*Zingiber officinale* Roscoe [Zingiberaceae; Zingiberis rhizoma recens]	Shengjiang	0.13	3.00
8^ac^	*Glycyrrhiza glabra* L. [Fabaceae; Glycyrrhizae radix et rhizome]	Gancao	1.50	9.00
9^c^	*Platycodon grandiflorus* (Jacq.) A.DC. [Campanulaceae; Platycodonis radix]	Jiegeng	3.00	9.00
10^c^	*Iris domestica* (L.) Goldblatt & Mabb. [Iridaceae; Belamcandae rhizome]	Shegan	1.50	9.00
11^d^	*Coptis chinensis* Franch. [Ranunculaceae; Coptidis rhizome]	Huanglian	0.35	3.00
12^d^	*Tetradium ruticarpum* (A.Juss.) T.G. Hartley [Rutaceae; Euodiae fructus]	Wuzhuyu	0.50	3.00
13	*Arcae concha* [main component: CaCO(3), (calcined)]	Duanwanglengzi	1.50	30.00
14	*Sepiae endoconcha* [main component: CaCO(3)]	Haipiaoxiao	1.50	20.00

^a^ 4 botanical drugs and 1 mineral drug included in Xuanfu Daizhe Decoction.

^b^ 5 botanical drugs included in Banxia Houpu Decoction.

^c^ 4 botanical drugs included in Gancao Jiegeng Shegan Decoction.

^d^ 2 botanical drugs included in Zuojin Wan.

THLY, Tonghua Liyan.

The aim of this study was to objectively evaluate the efficacy and possible adverse effects of THLY granules in the treatment of LPRD with stagnation of phlegm and qi syndrome (SPQS) and to formulate an effective Chinese medicine treatment plan for LPRD, which is a difficult disease in the clinic, to guide clinical practice.

## 2 Materials and methods

### 2.1 Study design and patients

This study was designed as a randomized, double-blind, placebo-controlled study based on standard therapy and parallel groups. All patients were LPRD patients attending Yueyang Hospital of Integrative Medicine, Shanghai University of TCM, and were screened according to the inclusion and exclusion criteria before entering the clinical trial study. The study conforms to the Declaration of Helsinki and was performed in accordance with Good Clinical Practice and national regulations. This study was reviewed by the Ethics Committee at Yueyang Hospital of Integrated Traditional Chinese and Western Medicine, Shanghai University of TCM (approval number: 2021–045), which was registered in the Chinese Clinical Trial Registry (ChiCTR2100046614). Written informed consent was obtained from each participant before the study was conducted. A withdrawal option was available to participants at any time during the study period.

LPRD was diagnosed based on the presence of typical symptoms (globus, throat clearing, hoarseness, chronic cough or dysphagia) and laryngoscopy findings. Eligible patients were aged between 18 and 75 years, had a positive reflux symptom index (RSI) (Belafsky et al., 2002) and reflux finding score (RFS) (Belafsky et al., 2001), had not used PPIs or prokinetic agents for 2 weeks before enrollment, and had TCM syndrome differentiated into SPQS. The differentiation criteria for SPQS were established in accordance with the *Consensus Opinion of Traditional Chinese Medicine Diagnosis and Treatment Experts on Gastroesophageal Reflux Disease (2017)* (Zhang et al., 2017). SPQS is a syndrome in TCM that manifests as a pharyngeal foreign body sensation, retrosternal discomfort, belching or reflux, dysphagia, hoarseness, midnight choking and coughing, white and greasy tongue coating, a string-like and slippery pulse and a series of other symptoms. The exclusion criteria were as follows: (a) had a combination of one of the following diseases: peptic ulcer, pyloric obstruction, Zollinger-Ellison syndrome, drug-induced esophagitis, primary esophageal dynamics, history of gastroesophageal and duodenal surgery, mycosis fungoides, or malignant tumors of the digestive tract; (b) had a combination of serious primary illnesses of other important systems that were not effectively controlled; (c) had severe psychiatric disease; (d) were in preparation for pregnancy, pregnant and lactating; (e) were allergic to the study drug or were taking other TCMs; (f) were unable to cooperate with the regular use of drugs and complete the data collection; and (g) had participated or were participating in other clinical trials 2 weeks before enrollment.

### 2.2 Sample size calculation

This test protocol is based on the test of difference between two sample rates, using a two-sided test, taking *α* = 0.05, *β* = 0.20, and PASS 15.0 to realize the sample calculation. The experimental group and the control group were allocated at a ratio of 1:1, and the main outcome was the clinical effectiveness rate. According to the previous literature, the efficacy rate of rabeprazole for the treatment of LPRD at week 8 was 57.98% (Lee et al., 2011). According to our preliminary study, the efficacy of THLY granules combined with rabeprazole for the treatment of LPRD was approximately 86.80%, and considering the 10% shedding rate, the total number of patients included was expected to be at least 76.

### 2.3 Quality control

**FIGURE 1:**
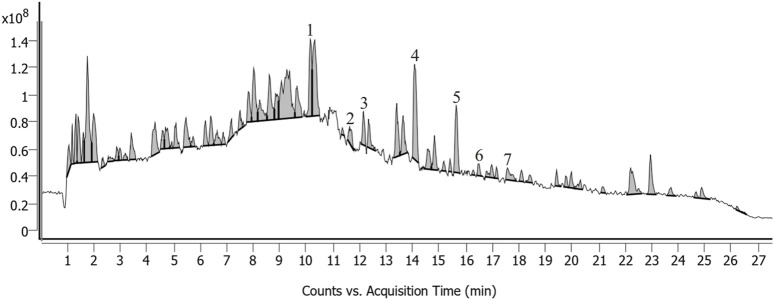
UPLC-QTOF-MS chromatograms of THLY granules. Peaks: Dihydrosanguinarine (1), Myristic acid (2), 1-O-Acetylbritannilactone (3), Glycyrrhizic acid (4), Irisflorentin (5), Glycycoumarin (6), Rutaecarpine (7).

Each pack of THLY granules was composed of 1.30 g of *Inula japonica* Thunb. [Asteraceae; Inulae flos], 0.43 g of *Haematitum* [main component: Fe (2)O (3), (calcined)], 2.80 g of *Pinellia ternata* (Thunb.) Makino [Araceae; Pinelliae rhizoma], 0.80 g of *Magnolia officinalis* Rehder & E.H. Wilson [Magnoliaceae; Magnoliae officinalis cortex], and 0.60 g of *Wolfiporia cocos* (F.A. Wolf) Ryvarden & Gilb. [Polyporaceae; Poria], 1.50 g of *Perilla frutescens* (L.) Britton [Lamiaceae] Perillae folium, 0.13 g of *Zingiber officinale* Roscoe [Zingiberaceae; Zingiberis rhizoma recens], 1.50 g of *Glycyrrhiza glabra* L. [Fabaceae; Glycyrrhizae radix et rhizome], 3.00 g of *Platycodon grandiflorus* (Jacq.) A. DC. [Campanulaceae; Platycodonis radix], 1.50 g of *Iris domestica* (L.) Goldblatt & Mabb. [Iridaceae; Belamcandae rhizome], 0.35 g of *Coptis chinensis* Franch. [Ranunculaceae; Coptidis rhizome], 0.50 g of *Tetradium ruticarpum* (A.Juss.) T.G. Hartley [Rutaceae; Euodiae fructus], 1.50 g of *Arcae concha* [main component: CaCO(3), (calcined)] and 1.50 g of *Sepiae endoconcha* [main component: CaCO(3)]. The THLY granule placebo consisted of THLY granules diluted to a 10% dosage with 90% excipients. The excipients were composed of 88.02% maltodextrin, 10% lactose, 1.32% edible caramel pigment, 0.03% edible sunset yellow pigment, 0.13% edible lemon yellow pigment and 0.5% bitter flavor. THLY granules were produced by Jiangyin Tianjiang Pharmaceutical Co., Ltd. (Number: 2103326). The qualified drugs were decocted with water and filtered to form a filtrate. The filtered solution was concentrated to a paste with a relative density of 1.00–1.13 (65°C ± 5 °C). The paste was then spray-dried, sieved and mixed to produce 12–40 mesh granules. The quality control of the THLY granules was performed according to the methods specified in the “Chinese Pharmacopoeia 2020.” All of the abovementioned crude drugs complied with quality inspection standards. Ultra-performance liquid chromatography with quadrupole time-of-flight mass spectrometry instrument (UPLC-QTOF-MS) was used to identify the components of THLY granules. The UPLC fingerprints show that the active ingredients of THLY granules include Dihydrosanguinarine, Myristic acid, 1-O-Acetylbritannilactone, Glycyrrhizic acid, Irisflorentin, Glycycoumarin, Rutaecarpine. Figure 1 and Supplementary Table S1 present the results of the quality analysis.

### 2.4 Randomization and blinding methods

The random sequence was generated by a professional statistician using SPSS 22.0 software. Drugs were uniformly packed and distributed in a random order. Patients were randomly assigned to the experimental or control group at a 1:1 ratio. The experimental group was given THLY granules (1 pack, twice daily) in combination with rabeprazole capsules (20 mg, once daily) for 8 weeks. The control group was administered the THLY granule placebo (1 pack, twice daily) combined with rabeprazole capsules (20 mg, once daily) for 8 weeks.

**FIGURE 2:**
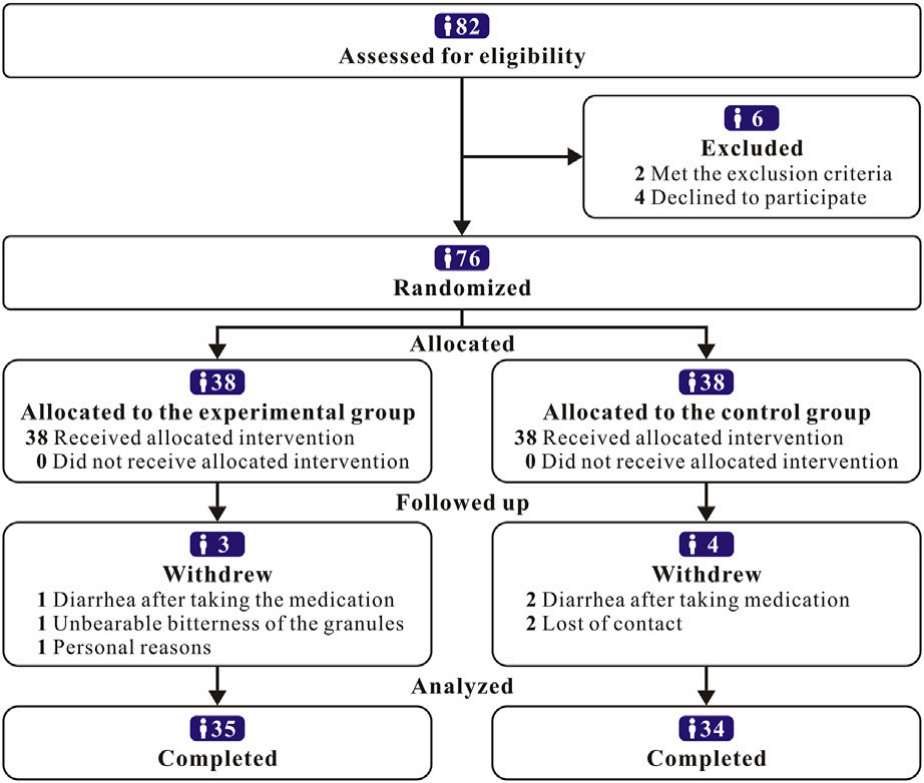
Flow diagram of the study population.

THLY granule formation was conducted in accordance with a double-blind design. The appearance, dosage form, and specifications of the THLY granules and THLY granule placebo were the same, and the outer packaging was printed with a number. Each drug sample was allocated an emergency letter as a decoder. A random key was sealed in duplicate in an envelope and given to the designated administrator. The patients, investigators, and anyone involved in the analysis were unaware of the trial drug class. The trial protocol is summarized in the flow diagram (Figure 2).

### 2.5 Outcome measures

The primary efficacy indicators included the RSI and clinical symptom score (Supplementary Table S2) at 8 weeks after randomization. The secondary efficacy indicators included the salivary pepsin concentration, RFS and gastroesophageal reflux disease questionnaire (GerdQ) at 8 weeks after randomization. Salivary pepsin was collected 1 hour after the meal. The patient was instructed to cough up 2–3 mL of saliva from deep within the throat, which was added to 0.5 mL of citric acid (0.1 mol/L) placed into a 5 mL saliva collection tube to avoid the inactivation of pepsin in the saliva. The pepsin concentration was detected with a human pepsin ELISA kit, and the absorbance (OD value) of the saliva samples was measured using an enzyme-linked immunosorbent assay. The sample concentration was calculated according to the OD value of the standard sample tested.

### 2.6 Safety evaluation indicators

Before treatment, the subjects’ sex, age, height, weight, course of disease, vital signs, medication history, family history, complications, etc., were recorded. Vital signs were observed and recorded after 8 weeks of treatment. Changes in safety indicators (including liver and kidney function before and after treatment) and adverse events that occurred during clinical research were observed and recorded.

### 2.7 Efficacy evaluation criteria

The RSI and clinical symptom score were used for efficacy evaluation. Efficacy was calculated as follows:

$$\text{efficacy}\text{value}=\left(\text{pretreatment}\text{score}‐\text{posttreatment}\text{score}\right)/\text{pretreatment}\text{score}\times 100\%.$$

Clinical recovery:Patients whose symptoms had disappeared and whose efficacy was≥95%;

Marked efficacy:symptoms mostly improved after treatment,and 70%≤efficacy value < 95%;

Efficacy:symptoms partially improved after treatment,and a 30%≤efficacy value < 70%;

Invalid:symptoms showed scarcely any improvement after treatment or were even worse,and the efficacy was <30%;

$$\text{overall efficacy rate}=\left(\text{clinical recovery}+\text{marked efficacy}+\text{efficacy}\right)\text{ cases}/a\text{ total number of cases}\times 100\%.$$

### 2.8 Statistical analysis

SPSS 22.0 software was used for statistical data processing and analysis. *p* < 0.05 indicated statistically significant differences, while *p* < 0.01 or *p* < 0.001 indicated highly statistically significant differences.

(1) Statistical description: Measurement data conforming to a normal distribution or approximate normal distribution are expressed herein as the mean ± standard deviation; data conforming to a skewed distribution are expressed as the median and upper and lower quartiles; count data or rank data are represented by the frequency, composition ratio, and rate.
(2) Statistical inference: For normally distributed or approximately normally distributed data, paired sample t tests were used for intragroup comparisons, and two independent sample t tests (with the same variance) or corrected t tests (with uneven variance) were used for intergroup comparisons. The Wilcoxon signed rank test was used for intragroup comparisons, and the Wilcoxon two-sample rank sum test was used for intergroup comparisons that conformed to a skewed distribution. The chi-square test or Fisher’s test was used for counting or grade data.

## 3 Results

### 3.1 Patient characteristics

A total of 76 LPRD patients with SPQS who met the diagnostic criteria of traditional Chinese and Western medicine were included in clinical trials, and 69 patients ultimately completed treatment. There were 3 patients in the experimental group who drpouted: 1 patient withdrew due to diarrhea after taking the medication, 1 patient withdrew due to unbearable bitterness of the granules, and 1 patient withdrew due to personal reasons. There were 4 patients in the control group who drpouted: 1 patient who withdrew due to diarrhea after taking the medication, 2 patients who lost contact, and 1 patient who withdrew for personal reasons. No significant differences were observed between the two groups regarding age, sex, body mass index (BMI) and course of disease (*p* > 0.05). The baseline gastroscopic classification was similar between the two groups (Table 2).

**TABLE 2:** Baseline characteristics.

Characteristics	Experimental group (n = 35)	Control group (n = 34)	t/χ^2^/Z	*p*-value
Gender				
Male	16 (45.71%)	14 (41.18%)		
Female	19 (54.29%)	20 (58.82%)	0.15	0.704
Age	52.03 ± 12.43	54.74 ± 11.37	−0.94	0.349
BMI (kg/m2)	22.24 ± 2.89	22.22 ± 3.34	0.03	0.975
Disease course	12 (5,24)	12 (6,27)	−1.42	0.156
Gastroscopic class				
NERD	19	18		
RE LA-A	14	15		
RE LA-B	1	0		
RE LA-C	0	0		
RE LA-D	1	0		
BE	0	1		

The values are expressed as the mean ± SD, or n (%) or M (P_25_, P_75_); BMI, body mass index; NERD, nonerosive reflux disease; RE, reflux esophagitis; BE, Barrett’s esophagus.

### 3.2 Primary outcomes

**FIGURE 3:**
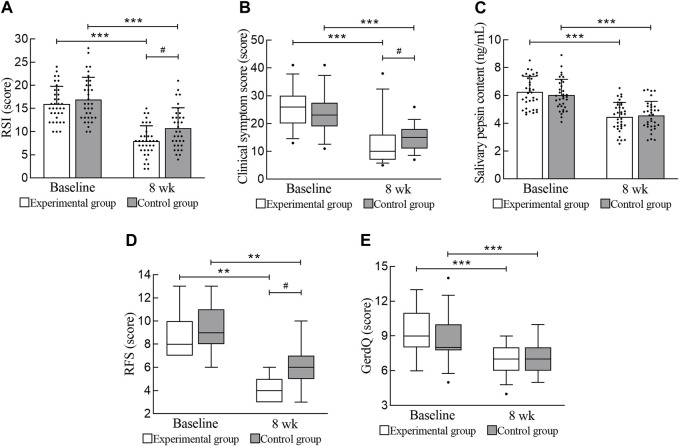
Primary and secondary efficacy outcomes. **(A)** RSI. **(B)** Clinical symptom score. **(C)** Salivary pepsin content. **(D)** RFS. **(E)** GerdQ. RSI, reflux symptom index; RFS, reflux finding score; GerdQ, gastroesophageal reflux disease questionnaire. *** Compared with baseline, *p* < 0.001; ** Compared with baseline, *p* < 0.01; # Compared with the experimental group, *p* < 0.05.

After treatment, both the experimental group and the control group showed significant improvements in the RSI and clinical symptom score (*p* < 0.001). The improvement in the RSI in the experimental group was higher than that in the control group (mean difference, 1.91 [95% CI, 0.12 to 3.70]; *p* = 0.037). The improvement in the clinical symptom score in the experimental group was higher than that in the control group (mean difference, 4.00 [95% CI, 1.00 to 7.00]; *p* = 0.011). The results are shown in Table 3 and Figure 3.

**TABLE 3:** Primary and secondary efficacy outcomes.

Outcomes	Visit	Mean ± SD or M (P_25_, P_75_)		t/Z	*p*-Value
		Experimental group (n = 35)	Control group (n = 34)		
Primary outcomes					
RSI	Baseline	15.97 ± 3.82	16.91 ± 4.81	−0.90	0.371
	8 weeks	7.91 ± 3.34	10.76 ± 4.38	−3.04	0.004
	Change	8.06 ± 3.55	6.15 ± 3.92	2.12	0.037
	(Mean difference, 1.91 [95% CI, 0.12 to 3.70])				
Clinical symptom score	Baseline	26.00 (20.00,30.00)	23.00 (19.00,27.50)	−1.20	0.229
	8 weeks	10.00 (7.00,16.00)	15.00 (11.00,18.00)	−2.39	0.017
	Change	12.00 (6.00,20.00)	7.50 (5.00,12.00)	−2.55	0.011
	(Mean difference, 4.00 [95% CI, 1.00 to 7.00])				
Secondary outcomes					
Salivary pepsin content	Baseline	6.26 ± 1.12	6.04 ± 1.12	0.84	0.405
	8 weeks	4.46 ± 1.05	4.55 ± 1.03	−0.37	0.713
	Change	1.81 ± 1.51	1.49 ± 1.58	0.86	0.395
	(Mean difference, 0.32 [95% CI, −0.42 to 1.06])				
RFS n = 15^a^	Baseline	8.00 (7.00,10.00)	9.00 (8.00,11.00)	−0.76	0.461
	8 weeks	4.00 (3.00,5.00)	6.00 (5.00,7.00)	−2.80	0.005
	Change	4.00 (4.00,6.00)	3.00 (3.00,4.00)	−2.64	0.010
	(Mean difference, 1.00 [95% CI, 0.00 to 2.00])				
GerdQ	Baseline	9.00 (8.00,11.00)	8.00 (7.75,10.00)	−1.15	0.250
	8 weeks	7.00 (6.00,8.00)	7.00 (6.00,8.00)	−0.01	0.990
	Change	2.00 (1.00,4.00)	2.00 (0.00,2.25)	−1.30	0.194
	(Mean difference, 1.00 [95% CI, 0.00 to 1.00])				

^a^ In terms of RFS, there were 15 patients in both the experimental and control groups.

### 3.3 Secondary outcomes

After treatment, the salivary pepsin content and GerdQ of the experimental group and the control group improved significantly (*p* < 0.001), but there was no significant difference between the two groups (*p* > 0.05). The effect of the experimental group in terms of reduced salivary pepsin content was not better than that of the control group. In this study, 15 laryngoscopic reexaminations were performed on both the experimental group and the control group, and the RFS significantly improved (*p* = 0.001). Moreover, the improvement in physical signs in the experimental group was significantly greater than that in the control group (mean difference, 1.00 [95% CI, 0.00 to 2.00]; *p* = 0.010). The results are shown in Table 3 and Figure 3.

### 3.4 Outcome assessment for efficacy evaluation

In terms of the RSI, the experimental group had 5 cases of marked efficacy, 25 cases of efficacy, and 5 cases of invalidity, for an overall efficacy rate of 85.71%. The control group had 1 case of marked efficacy, 19 cases of efficacy, and 14 cases of invalidity, for an overall efficacy rate of 58.82%. There was a statistically significant difference in the overall efficacy rate between the experimental group and the control group in terms of the RSI (*p* = 0.012). In terms of the clinical symptom score, the experimental group had 8 marked efficacy cases, 20 efficacy cases, and 7 invalid cases, for an overall efficacy rate of 80.00%. The control group had 2 marked efficacy cases, 16 efficacy cases, and 16 invalid cases, for an overall efficacy rate of 52.94%. There was a statistically significant difference in the overall efficacy rate between the experimental group and the control group in terms of the clinical symptom score (*p* = 0.017). In summary, the treatment effect in the experimental group was greater than that in the control group (Table 4).

**TABLE 4:** Outcome assessment for efficacy evaluation.

Outcomes	Visit	n (%)		*χ* ^2^	*p*-Value
		Experimental group (n = 35)	Control group (n = 34)		
RSI	Overall efficacy	30 (85.71%)	20 (58.82%)	6.25	0.012
	Invalid	5 (14.29%)	14 (41.18%)		
Clinical symptom score	Overall efficacy	28 (80.00%)	18 (52.94%)	5.68	0.017
	Invalid	7 (20.00%)	16 (47.06%)		

Overall efficacy = clinical recovery + marked efficacy + efficacy.

### 3.5 Safety evaluation

During the study, the liver and kidney functions of the patients before and after treatment were tested. There was no obvious abnormality, and no drug allergy reaction was found. One patient in the experimental group developed diarrhea after cholecystectomy, with mild reactions; one patient in the control group experienced diarrhea.

## 4 Discussion

LPRD is the reflux of gastric contents to the upper esophageal sphincter (UES), reaching the throat, oropharynx and other parts. Reflux stimulates the throat mucosa, causing a foreign body sensation, sore throat, throat clearing and other symptoms, often coexisting with typical GERD. LPRD, a manifestation of GERD’s extraesophageal syndrome, has basically the same pathogenesis as GERD. Studies have shown that LPRD is related mainly to acid reflux, indirect stimulation of the vagus nerve reflex, nocturnal acid breakthrough, visceral hypersensitivity, and CYP2C19 gene polymorphism. Several studies have shown that the presence of H+/K + -ATPase in the throat may induce acid production and cause mucosal damage (Becker et al., 2015). The treatment principle for LPRD in modern medicine is to alleviate symptoms and improve patient quality of life. Commonly used drugs include PPIs and potassium competitive acid blockers (P-CABs) (Iwakiri et al., 2022).

Currently, LPRD treatment relies mainly on the RSI and/or RFS for initial diagnosis. In this study, LPRD patients were significantly lower in the RSI and RFS after 8 weeks of treatment. THLY granules combined with PPIs improved symptoms more significantly, with a higher overall efficacy rate. This combined treatment significantly improved the symptoms and signs in the throat and stomach, improved the clinical efficacy of LPRD, and improved the patient’s throat and stomach discomfort.

Salivary pepsin detection is currently the simplest diagnostic method for LPRD and is fast, inexpensive and noninvasive. However, further research is still needed on the determination method, determination time and diagnostic threshold. Salivary pepsin detection methods, including ELISA, Western blotting, fibrinogen lysis and the Peptest, have continually emerged. The existing studies use different detection methods, reagent manufacturers and production batch numbers, resulting in different detection results (Jing et al., 2023). Furthermore, saliva dilution and esophageal clearance may lead to low concentrations of pepsin that cannot be detected. Therefore, there is no international unified detection standard for salivary pepsin content. After 8 weeks of treatment, both the experimental group and the control group in this study exhibited a reduced concentration of pepsin in saliva. When THLY granules were combined with PPIs, the concentration of pepsin in saliva decreased, but the difference between the two groups was not statistically significant (*p* > 0.05), which may be related to the lower baseline level of pepsin in the saliva of LPRD patients, the inaccurate sampling time points, and salivary dilution. The amount of saliva retained should be increased to help assess the actual saliva pepsin level.

THLY granules are prescribed by the “Tong, Hua, Xuan, Ping” Differentiation and Treatment System of Ding’s Internal Medicine Chen Cunren Academic Ideology Research Base of Shanghai style TCM. It is derived from Xuanfu Daizhe Decoction, Banxia Houpu Decoction, Gancao Jiegeng Shegan Decoction, and Zuojin Wan. Xuanfu Daizhe Decoction (Yang et al., 2021), Pinellia Tuber, Golden Thread and Medicinal Evodia Fruit present in this recipe can inhibit the secretion of 5-hydroxytryptamine in the chromaffin cells of the gastrointestinal mucosa and inhibit the esophageal bronchial nerve reflex caused by vagus nerve C fibers (Yang, 2013). Second, Banxia Houpu Decoction can regulate the TRP pathway and arachidonic acid metabolism (Zhao et al., 2020) and reduce IL-6 and TNF-α levels (Tang, 2021). In addition, Banxia Houpu Decoction can improve esophageal mucosal inflammation, inhibit laryngeal reflex activity, and alleviate globus symptoms in patients by inducing PGE2 and PGI2 to inhibit the secretion of gastric acid (Guan et al., 2021). Moreover, Gancao Jiegeng Shegan Decoction can regulate the levels of cyclooxygenase-2 and PGE2 (Yang et al., 2017); reduce the serum TNF-α, IL-1β, and IL-6 levels (Huang et al., 2020); reduce throat inflammation; and exert expectorant effects. Network pharmacology data suggest that Zuojin Wan influences key proteins, such as TNF-α, IL6, ERK, p38, and JNK, which regulate the inflammatory response and participate in cell proliferation and apoptosis processes (Wei et al., 2021).

LPRD patients with SPQS have unremarkable symptoms of acid reflux and heartburn, which may lead to fewer types of acid reflux and cause less pronounced esophageal inflammation; therefore, the endoscopic presentations are mostly nonerosive reflux disease (NERD) and reflux esophagitis (RE) LA-A. Jonaitis L et al. reported that patients with LPRD had 67% NERD and 33% esophagitis. Seventy-two percent of esophagitis occurred in the LA-A group (Jonaitis et al., 2006).

The main pathogenesis of LPRD with SPQS is “upward perversion of stomach qi, phlegm stagnation of throat”. This study confirmed that THLY granules reduce throat inflammation and inhibit reflux in patients, especially when they cause symptoms of pharyngeal foreign body sensation and cough with sputum. This effect is related to the pharmacological components of TCM, which have anti-inflammatory and analgesic effects, inhibit gastric acid secretion, promote gastric peristalsis, and repair mucosal damage. It is speculated that THLY granules can regulate inflammatory factors via multiple targets; participate in cell proliferation and apoptosis; inhibit bile, pepsin, trypsin and other nonacid reflux substances; compensate for the deficiency of PPIs in treating nonacid reflux LPRD; and provide a theoretical basis for the use of PPIs combined with TCM to treat LPRD.

## 5 Conclusion

THLY granules combined with PPIs can improve the clinical symptoms and signs of LPRD assessed by laryngoscopy, reduce the salivary pepsin concentration, and inhibit the reflux of gastric contents. THLY granules combined with PPIs have a better effect than PPIs alone. THLY granules are safe and effective, have no obvious adverse reactions, and are worthy of clinical promotion and further in-depth research.

## Data Availability

The original contributions presented in the study are included in the article/Supplementary Material, further inquiries can be directed to the corresponding authors.
