# Supplementary material for: Tonghua Liyan granules in the treatment of Laryngopharyngeal reflux disease with stagnation of phlegm and qi syndrome: a randomized, double-blind, placebo-controlled study
Source: Front Pharmacol. 2024 Feb 13;15:1275740. doi: 10.3389/fphar.2024.1275740 (PMC10921225; doi:10.3389/fphar.2024.1275740)
Supplement: Supplementary file 1 [file Table1.DOCX]

**Supplementary Table S1** Identification of the constituents of THLY granules

| Peak | Component | Time (min) | Formula | Area | Mass | CAS | Source |
| --- | --- | --- | --- | --- | --- | --- | --- |
| 1 | Dihydrosanguinarine | 10.151 | C20 H15 N O4 | 371120267 | 333.1005 | 3606-45-9 | *Coptis chinensis* Franch. [Ranunculaceae; Coptidis rhizome] |
| 2 | Myristic acid | 11.630 | C14 H28 O2 | 107453128 | 228.2093 | 544-63-8 | *Platycodon grandiflorus* (Jacq.) A.DC. [Campanulaceae; Platycodonis radix] |
| 3 | 1-O-Acetylbritannilactone | 12.149 | C17 H24 O5 | 131551950 | 308.1629 | 681457-46-5 | *Inula japonica* Thunb. [Asteraceae; Inulae flos] |
| 4 | Glycyrrhizic acid | 14.075 | C42 H62 O16 | 564629162 | 822.4040 | 1405-86-3 | *Glycyrrhiza glabra* L. [Fabaceae; Glycyrrhizae radix et rhizome] |
| 5 | Irisflorentin | 15.641 | C20 H18 O8 | 329072057 | 386.1002 | 41743-73-1 | *Iris domestica* (L.) Goldblatt & Mabb. [Iridaceae; Belamcandae rhizome] |
| 6 | Glycycoumarin | 16.468 | C21 H20 O6 | 51989134 | 368.1260 | 94805-82-0 | *Perilla frutescens* (L.) Britton [Lamiaceae] Perillae folium |
| 7 | Rutaecarpine | 17.571 | C18 H13 N3 O | 102006477 | 287.1063 | 84-26-4 | *Tetradium ruticarpum* (A.Juss.) T.G. Hartley [Rutaceae; Euodiae fructus] |
