# Supplementary material for: Tonghua Liyan granules in the treatment of Laryngopharyngeal reflux disease with stagnation of phlegm and qi syndrome: a randomized, double-blind, placebo-controlled study
Source: Front Pharmacol. 2024 Feb 13;15:1275740. doi: 10.3389/fphar.2024.1275740 (PMC10921225; doi:10.3389/fphar.2024.1275740)
Supplement: Supplementary file 2 [file Table2.DOCX]

**Supplementary Table S2** Clinical symptom scores

| Main symptom (score) | Normal (0) | Mild (2) | Moderate (4) | Severe (6) |
| --- | --- | --- | --- | --- |
| Pharyngeal foreign body sensation | No | Occasional discomfort in the throat, which can be relieved by oneself | Occasional discomfort in the throat, not affecting work and rest | Persistent discomfort in the throat, noticeable and unbearable |
| Retrosternal discomfort | No | Occasional chest pain with a short onset time | Occasional chest pains of long duration | Frequent chest pains that last long enough to interfere with work and rest |
| Throat clearing | No | Occasional mild throat clearing, 1-2 times per day | Effortful throat clearing, 3-10 times per day | Frequent throat clearing, >10 times per day |
| Pharyngeal itching and sore throat | No | Occasional itchy sore throat, which can only be felt with attention | Frequent itchy sore throat, tolerable | Significant itchy and sore throat, affecting life activities and often requiring medication |
| Heartburn | No | Occasionally, <1 time per day | Occurring at times, 1-3 times per day | Frequent, affecting work and rest |
| Acid reflux | No | Occasionally, <1 time per day | Occurring at times, 1-3 times per day | Frequent, affecting work and rest |
| Secondary symptoms | Normal (0) | Light (1) | Moderate (2) | Severe (3) |
| Belching or hiccups | No | Occasional, <4 times per day | Occurring at times, 4-10 times per day | Frequent, affecting work and rest |
| Dysphagia | No | Occasional, self-resolving | Occurring at times, relieved by medication | Frequent, not relieved by medication, interferes with eating |
| Hoarseness | No | Occasional, <1 time per day | Occurring at times, 1-3 times per day | Frequent, affecting work and rest |
| Midnight choking and coughing | No | Occasional, <1 time per week | Occurring at times, 1-3 times per week | Frequent, affecting rest |
| Cough with sputum | No | There is a slight cough with a small amount of mucus in the throat | Cough with visible mucus in the throat | Troublesome cough and phlegm, affecting work and rest |
| Stomach fullness | No | Occasional, slight stomach fullness | Stomach fullness is occasional and tolerable | Frequent stomach bloating that interferes with work and rest |
| Nausea and retching | No | Occasional, <1 time per day | Occurring at times, 1-3 times per day | Frequent, affecting work and rest |
| Sticky and greasy mouth | No | Occasional, <1 time per day | Occurring at times, 1-3 times per day | Frequent, affecting work and rest |
| Insomnia | No | Occasional | Moderate | Prolonged and persistent insomnia that interferes with work and rest |
| Emotional disorders | No | Occasional | Moderate | Significant emotional imbalance that interferes with work and rest |
